# Long-Term Evolution of Skeletal Muscle Quantity and Quality After Curative-Intent Colon Cancer Surgery: A Retrospective Cohort Study †

**DOI:** 10.3390/diagnostics15233092

**Published:** 2025-12-04

**Authors:** Argyri Papadimitriou, Michael Schneider, Salim Zenkhri, Dieter Hahnloser, David Martin, He Ayu Xu, Damien Maier, Fabio Becce, Fabian Grass, Martin Hübner

**Affiliations:** 1 Faculty of Biology and Medicine, University of Lausanne (UNIL), 1011 Lausanne, Switzerland; argyri.papadimitriou@unil.ch; 2 Department of Visceral Surgery, Lausanne University Hospital (CHUV), University of Lausanne (UNIL), 1011 Lausanne, Switzerland; 3 Department of Diagnostic and Interventional Radiology, Lausanne University Hospital (CHUV), University of Lausanne (UNIL), 1011 Lausanne, Switzerland; 4 Biomedical Data Science Center, Lausanne University Hospital (CHUV), University of Lausanne (UNIL), 1011 Lausanne, Switzerland

**Keywords:** colorectal surgery, computed tomography-based sarcopenia, sarcopenia long-term follow-up, muscle quantity, muscle quality, survival

## Abstract

**Background**: Computed tomography (CT)-based sarcopenia is a promising predictor of postoperative complications and recovery. However, studies on the longitudinal evolution of skeletal muscle markers are lacking and findings regarding its correlation with survival are still not clear. **Methods**: We conducted a retrospective single-center cohort study of consecutive patients undergoing curative-intent colon cancer surgery. Skeletal muscle area (SMA), skeletal muscle index (SMI), skeletal muscle radiation attenuation (SMRA), and intermuscular adipose tissue (IMAT) area and index (IMATI) were measured on a single axial CT slice at the third lumbar vertebral level before surgery and at 6, 12, and 24 months after. Descriptive statistics were used to report the evolution over time of CT-based sarcopenia markers. Their correlation with overall survival was analyzed using Cox’s proportional hazards regression analysis. **Results**: The final cohort included 102 patients (65.7% males) with a mean age of 66 ± 13 years. Eighty-five (86.7%) patients were alive at 24 months, and forty-five (45.9%) underwent a CT scan at all time points. CT-based sarcopenia markers remained statistically stable over 2 years. Age (HR 1.07, 95% CI 1.00–1.14) and ASA score (HR 2.4, 95% CI 1.00–5.7) were negative independent predictive factors. Patients with larger differences (Δ) of IMAT area and IMATI at 12 months, HR 0.79 (95% CI 0.67–0.93) and 0.49 (95% CI 0.30–0.80), respectively, had a lower mortality. **Conclusions**: CT-based markers of skeletal muscle quantity (SMA, SMI) and quality (IMAT area, IMATI) remained statistically stable after curative-intent colon cancer surgery. No preoperative CT-based sarcopenia markers were predictive of overall survival. Larger cohorts are needed to generalize these initial findings.

## 1. Introduction

Cancer patients may be exposed to sarcopenia, which has been increasingly recognized as a surrogate marker of vulnerability, frailty, and malnutrition [1,2]. Since 2016, the International Classification of Diseases (ICD-10) has officially endorsed sarcopenia as a muscle disease. Two recent systematic reviews and meta-analyses revealed prevalence rates between 5 and 17% [3] and 10–27% [4], respectively. Sarcopenia is an age-related disease, and cancer patients are even more exposed due to anorexia, metabolic alterations, and inflammation. The prevalence in patients with colorectal cancer (CRC) varies between 12% and 60% [5]. Sarcopenia can be assessed on a CT scan at a specific lumbar vertebral level (e.g., L3) by measuring muscle quantity markers—skeletal muscle area (SMA) and skeletal muscle index (SMI)—and quality markers—skeletal muscle radiation attenuation (SMRA, a measure of intramyocellular fat)—as well as the presence of subfascial, extramyocellular adipose tissue—intermuscular adipose tissue (IMAT) area and index (IMATI). CT-based sarcopenia has been correlated with postoperative complications after oncological colon resection [6]. Most studies found it to be a predictor of worse perioperative and oncological outcomes. However, there is significant heterogeneity in the definition of sarcopenia used in previous studies. Moreover, these studies focused on the presence or absence of sarcopenia at a given time point, relying on predefined cutoffs [7,8,9,10]. To our knowledge, this is the first study to demonstrate the evolution of CT-based sarcopenia markers over time, at three different time points after oncological colon surgery without using cutoffs defined in specific populations.

## 2. Materials and Methods

### 2.1. Patients

**Figure 1:**
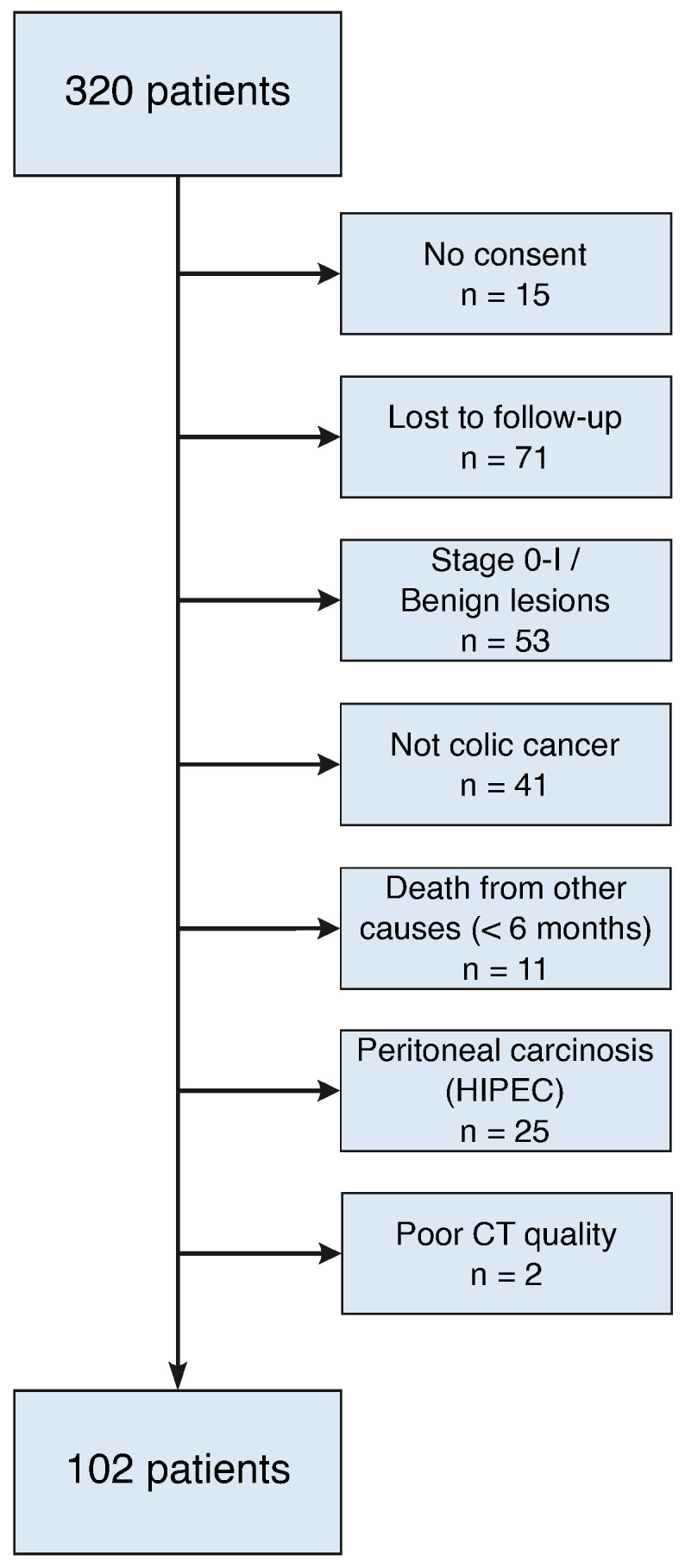
Flow diagram with exclusion criteria.

This single-center retrospective cohort study included consecutive patients who underwent oncological colon resections at the Department of Visceral Surgery at Lausanne University Hospital (CHUV) between January 2014 and December 2019. Exclusion criteria included insufficient/incomplete follow-up with CT imaging, benign lesions or stage 0-I tumor (no systematic follow-up with CT), cytoreductive surgery and hyperthermic intraperitoneal chemotherapy (HIPEC), and lack of informed consent for study participation (Figure 1). All patients were treated according to the institutional Enhanced Recovery After Surgery (ERAS) protocol for colon surgery [11,12] and the Swiss Society of Gastroenterology guidelines [13]. This study was carried out according to the Declaration of Helsinki and was approved by the *Commission cantonale d’éthique de la recherche sur l’être humain* (CER-VD, protocol no. 2020-00677). Demographic data, comorbidities, tumor stage [14,15], surgical specifics, and postoperative complications were evaluated as previously described.

### 2.2. CT-Based Body Composition Assessment

The different muscle quantity and quality markers (SMA, SMI, SMRA, IMAT area, and IMATI) were measured at four different time points: preoperatively, 6, 12, and 24 months after surgery. For patients treated with neoadjuvant chemotherapy, we used CT **scans obtained after treatment. The vast majority of CT scans were performed using the** institution’s standardized CT protocol (same CT manufacturer (GE Healthcare), 120 kVp tube potential) after intravenous contrast medium administration (portal venous phase) at predefined follow-up time points. Single axial CT slices were extracted from the Picture Archiving and Communication System (PACS) in the Digital Imaging and Communications in Medicine (DICOM) format and included the psoas, paraspinal, and abdominal wall muscles at the third lumbar vertebral level (mid-pedicle). Muscle quantity and quality markers were measured using a semiautomated deep learning-based algorithm with a U-Net architecture [16,17]. As in previous studies using the same algorithm [18,19,20], all deep learning muscle segmentations were then reviewed and corrected as appropriate using a custom graphical user interface by a board-certified musculoskeletal radiologist. To assess muscle quantity, SMA was measured in ${\mathrm{cm}}^{2}$ and normalized by the patient’s height squared to obtain the SMI in ${\mathrm{cm}}^{2}$/$m^{2}$. SMRA was expressed in Hounsfield units (HUs) to estimate muscle quality based on its density. The IMAT area assessed the subfascial, extramyocellular fat pixels located between muscles and was normalized by the patient’s height squared to obtain the IMATI in ${\mathrm{cm}}^{2}$/$m^{2}$. SMRA and IMAT area were obtained by distinguishing within SMA the pixels that attenuated as muscle from those that attenuated as adipose tissue using standard HU thresholds: −29 to +150 HU for skeletal muscle, and −190 to −30 HU for adipose tissue [21]. SMRA and IMAT area/IMATI can be used as markers of intramyocellular and extramyocellular steatosis, respectively [22].

### 2.3. Statistical Analysis

Continuous data were presented as mean (SD) and compared with the Student’s *t*-test. Categorical variables were presented as frequencies (%) and analyzed with Pearson’s chi-squared or Fisher’s exact test, as appropriate. SMA, SMI, SMRA, IMAT area, and IMATI mean values were computed separately for each sex at each time point and compared on graphs using Python (pandas 2.0.3 and seaborn 0.13.2). Subsequently, groups were created with respect to chemotherapy treatment and oncological outcome within the observation period of 24 months. The evolution of CT-based sarcopenia markers of these groups was compared using the same type of graphics. The difference (Δ) in SMI, SMA, SMRA, IMAT area and IMATI between the preoperative mean and the mean value at each time point was also calculated.

Then, univariate Cox’s proportional hazards regression analyses were used to determine whether preoperative values of muscle quantity and quality, or their difference over time, were correlated with overall survival. Hazard ratios (HRs) reflect the change in risk per one-unit increase in each predictor variable, in their original measurement units (no standardization or scaling was applied). Continuous predictors were modeled as linear, untransformed variables. Missing data were handled using complete case analysis, such that individuals with missing values for any variable included in a given model were excluded. Participants who had not died at the end of follow-up, or who were lost to follow-up, were right-censored at the time of last contact. A *p*-value ≤ 0.05 was considered statistically significant. The proportional hazards assumption was validated for all indicators using Schoenfeld’s residual-based diagnostics at *p* < 0.05. All analyses were performed using scikit-learn (1.0.2) and scipy (1.10.1).

## 3. Results

The final study cohort included 102 eligible patients. Table 1 details their demographics, tumor specifics, and preoperative sarcopenia measurements.

**Figure 2:**
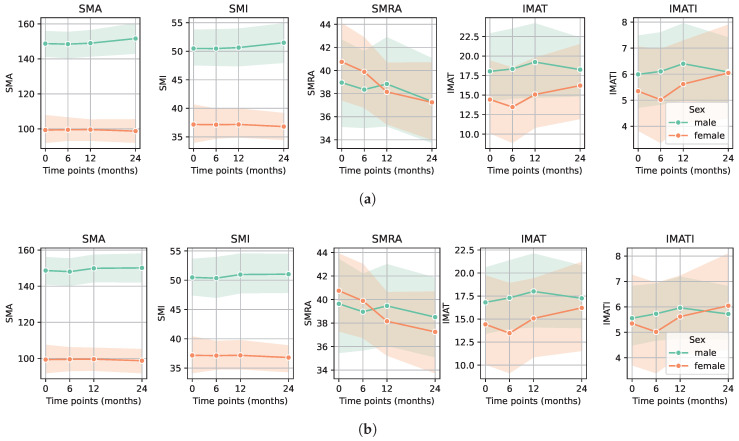
Evolution of CT-based sarcopenia markers for each sex. (**a**) Sarcopenia markers in males and females before surgery (time point 0) and postoperatively at 6, 12, and 24 months for the entire cohort showed a decrease in SMRA (*p*-value: 0.549 for males and 0.179 for females). (**b**) Sarcopenia markers in males and females before surgery (time point 0) and postoperatively at 6, 12, and 24 months for the surviving patients who had a CT at every time point showed again a decrease in SMRA (*p*-value: 0.667 for males and 0.179 for females).

The evolution of CT-based sarcopenia markers is displayed by sex for the entire cohort (Figure 2a). Markers remained stable over time except for SMRA, which numerically decreased for both sexes; however, it did not reach statistical significance. The mean overall survival during the study period was 22 ± 5.3 months. Eighty-five patients (86.7%) were alive at 24 months and forty-five (45.9%) had a CT scan at all four time points (CT scan not performed routinely at 6 months follow-up). SMRA was the only marker that decreased over time in these patients, again without reaching statistical significance: mean ΔSMRA at 24 months was −1.1 (SD 5.3, *p*-value: 0.667) in males and −3.5 (SD 4.5, *p*-value: 0.179) in females. The evolution of all sarcopenia markers for the surviving patients is depicted in Figure 2b.

**Figure 3:**
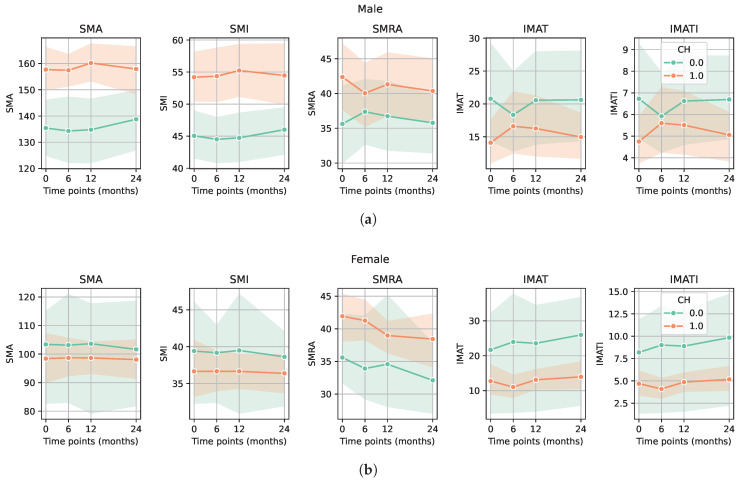
Evolution of CT-based sarcopenia markers in patients treated with chemotherapy compared to patients who did not receive chemotherapy. (**a**) Male patients treated with chemotherapy had higher values of SMA/SMI and SMRA and lower values of IMAT area/IMATI (*p*-value < 0.05 for all markers). (**b**) Female patients treated with chemotherapy had higher values of SMRA and lower values of IMAT area/IMATI. *p*-value < 0.05 for SMRA, IMAT area, and IMATI. Among female patients, we did not observe a significant difference between those who received chemotherapy and those who did not, in contrast to male patients, possibly due to the smaller sample size of female patients.

**Figure 4:**
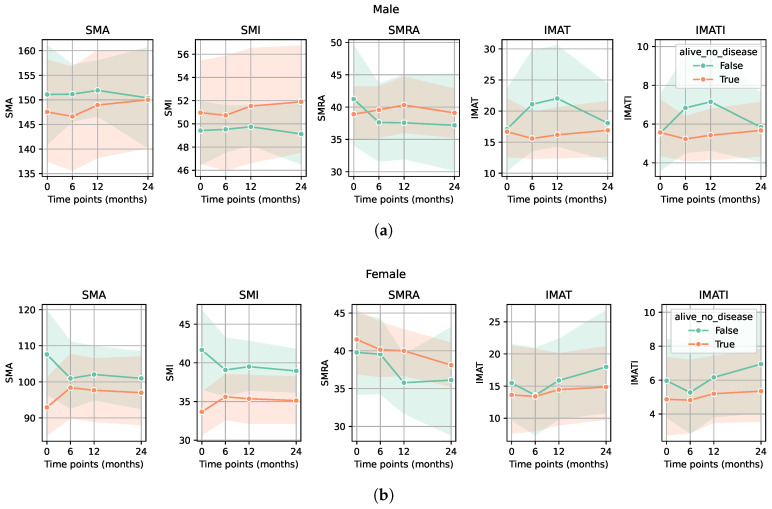
Evolution of CT-based sarcopenia markers for patients in remission compared to all other patients (surviving patients with recurrence and deceased patients). There were no significant differences in either sarcopenia marker between the group of patients in remission and all other patients for either male (**a**) or female (**b**) groups (*p*-values > 0.05).

In the group of patients treated with chemotherapy, preoperative or adjuvant, the SMA, SMI, and SMRA values were significantly higher compared with the patients without chemotherapy. In the same group, IMAT area and IMATI values were significantly lower (*p*-value < 0.05 for all markers) (Figure 3a,b). Regarding the group of patients in remission, no significant differences in sarcopenia markers were observed compared to all others (patients with recurrence, progression, or deceased) (Figure 4a,b).

**Figure 5:**
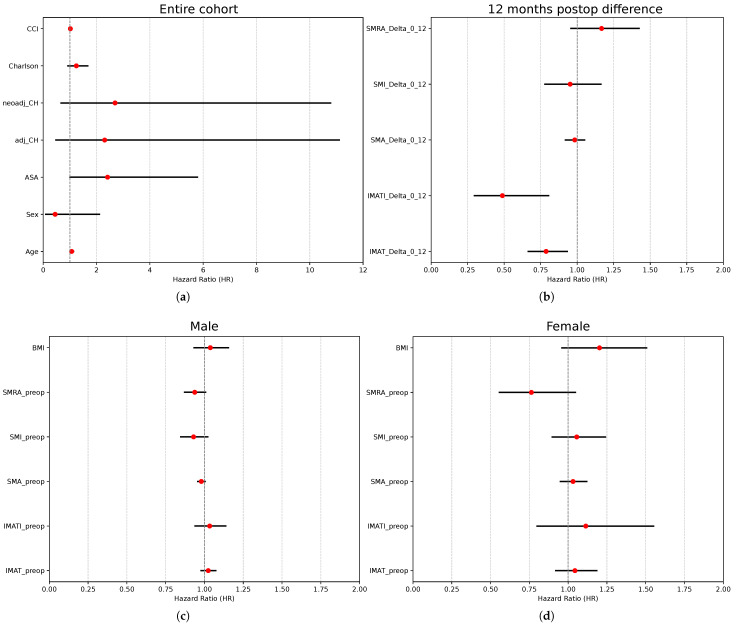
Prognostic factors for overall survival after curative-intent colon surgery. (**a**) Cox’s regression analysis of independent variables—entire cohort. (**b**) Cox’s regression analysis of difference in sarcopenia markers 1 year after surgery vs. preceding surgery—entire cohort. (**c**) Cox’s regression analysis of preoperative sarcopenia markers in male patients. (**d**) Cox’s regression analysis of preoperative sarcopenia markers in female patients.

The univariate Cox regression analysis revealed age (HR 1.07, 95% CI: 1.00–1.14) and ASA score (HR 2.4, 95% CI: 1–5.7) as negative predictive values for overall survival (Table 2, Table 3 and Table 4 and Figure 5). None of the sarcopenia markers at baseline were predictive of overall survival. Patients with higher Δ values of IMAT area and IMATI at 12 months after surgery (meaning with a more significant increase in IMAT area/IMATI 12 months after surgery) had a lower risk of death–HR: 0.79 (CI: 0.67–0.93)/0.49 (CI: 0.30–0.80).

## 4. Discussion

To our knowledge, this is the first study to evaluate the dynamics of radiological sarcopenia markers after oncological colon surgery at different time points. In our cohort, SMA/SMI and IMAT area/IMATI remained largely stable at 24 months. We only observed a numerical decrease in SMRA until 24 months after surgery in both men and women; however, this was not statistically significant. It is important to bear in mind that muscle quantity decreases with age when interpreting the results. Consequently, the underlying causes could be related to aging, medical treatment, myosteatosis, or a combination of all the above. Interestingly, SMRA was also lower in patients with major complications and prevailed over muscle quantity in predicting short-term adverse outcomes after oncological colon surgery in a previous study of our group [6]. The fact that SMRA had a decreasing trend compared with the other parameters, while being more strongly correlated with postoperative complications raises the hypothesis that muscle quality is a key parameter to study and attempt to improve with rehabilitation programs. More long-term analyses of muscle quality are needed to confirm the trend toward decreased muscle quality.

Several studies demonstrated that preoperative sarcopenia was associated with a decrease in overall survival (OS), recurrence-free survival (RFS), and colorectal cancer-specific survival (CSS) [7]. As described in the study by Hopkins et al. [8], muscle quality and quantity markers were associated with poorer OS, RFS, and CSS. At the same time, BMI was not a significant factor in the same cohort of 968 patients with stage I to III CRC undergoing curative resection, emphasizing that body composition may be more reliable than BMI, which is important in an era where obesity prevalence is increasing. Sarcopenia has also been analyzed as a risk factor for prolonged LOS [23]. The fact that the present study did not demonstrate an association between preoperative sarcopenia markers and survival is either due to the smaller sample size or to its different methodology. Different methods are commonly used to assess sarcopenia in practice and research. The validated tests and tools are well described by the EWGSOP2 group. CT scan assessment to measure sarcopenia markers on a single CT slice at the L3 vertebral level represents a validated tool [24]. The measures are objective and reliable and can easily be obtained on CRC patients’ routine follow-up CT-scans without adding radiation burden or supplementary exams. Machine learning further provides a reproducible and reliable method for assessing CT-based sarcopenia. In order to define sarcopenia, different thresholds have been proposed [1,9,25,26]. For SMI, a cutoff value of 52.4 ${\mathrm{cm}}^{2}$/$m^{2}$ for male patients and 38.5 ${\mathrm{cm}}^{2}$/$m^{2}$ for female patients has been suggested. Nevertheless, these thresholds should be used with caution because they depend on ethnicity and the underlying disease. Moreover, cutoffs, which are determined in heterogeneous populations, cannot necessarily be applied to a specific group of patients like surgical colon cancer patients. For this reason, we chose to study the trend of each muscle marker and compare them between the different groups rather than using predefined cutoffs. This is a novelty and one of the strengths of our study.

Another interesting aspect of studying sarcopenia in oncological patients is understanding its association with chemotherapy. A systematic review showed that chemotherapy-induced toxicities were significantly associated with low skeletal muscle mass in non-metastatic CRC patients [27]. In the present study, both muscle quantity and quality were significantly higher in chemotherapy patients than in patients who did not receive any chemotherapy. In addition, they presented with less muscular fat infiltration. This could be explained by the fact that this group of patients was followed more closely with concomitant nutritional monitoring and treatment. On the other hand, the difference in the IMAT area and IMATI in these groups could raise the hypothesis that chemotherapy agents are more responsible for fat than muscle loss. In the current literature, there is a discrepancy concerning the changes in body composition due to chemotherapy, but a prospective study of patients with urothelial carcinoma also found that chemotherapy was responsible for fat loss rather than lean mass loss [28]. Two studies on breast cancer patients found that different chemotherapeutic agents were responsible for either the gain of fat mass or fat-free mass and that the lean mass of patients treated with chemotherapy significantly increased in the first 6 months after the treatment [29,30]. With the expansion of machine learning and artificial intelligence, radiological markers of sarcopenia and deltas between different time points may become an integral part of standard follow-up, helping clinicians adapt both chemotherapy doses and nutritional therapy. Currently, these elements are adapted to the body surface area (BSA) rather than to the body composition [31,32,33].

This study has limitations related to its retrospective design and limited sample size (type 2 error). The Cox regression analysis showed that patients with higher Δ values of the IMAT area and IMATI at 12 months after surgery may have a lower risk of death. This rather counterintuitive finding could be due to the limited sample size of patients and the overall low mortality. Because only a subset of participants completed all medical examinations at all time points, longitudinal analyses and subsequent survival associations could include selection bias, as patients with full follow-up may represent a healthier cohort than those with incomplete data. Consequently, longitudinal estimates should be interpreted with caution.

In addition, although the vast majority of CT scans were performed in the institution using a standardized CT protocol, a few of them were performed in nearby centers, which creates some heterogeneity in the imaging dataset. However, all CT scans and muscle segmentations were reviewed and corrected by an expert musculoskeletal radiologist. Other potential confounders may be related to lifestyle changes, different indications, and the duration of chemotherapy (fitter patients are more likely to receive chemotherapy).

In conclusion, CT-based sarcopenia markers and their dynamics did not appear to predict oncological outcomes. Larger cohorts should confirm these initial results for further generalization. More research should focus on the potential role of sarcopenia in guiding nutritional and physical exercise interventions in the perioperative setting and during the long-term follow-up of oncological patients.

## Figures and Tables

**Table 1:** Baseline characteristics of the study population. Numbers are reported as mean (±SD) or n (%); BMI: body mass index; CCI: comprehensive complication index; SMA: skeletal muscle area; SMI: skeletal muscle index; SMRA: skeletal muscle radiation attenuation; IMAT: intermuscular adipose tissue; IMATI: IMAT index.

Overall Population (n = 102)	Male (n = 67)	Female (n = 35)	*p*-Value
Alive	53 (79.1)	32 (91.4)	
without disease	39 (58.2)	23 (65.7)	
with disease	14 (20.9)	9 (25.7)	
Death	14 (20.9)	3 (8.6)	
due to the disease	11 (16.4)	3 (8.6)	
due to other cause	3 (4.5)		
Age	66.2 (13.3)	65.9 (13.5)	0.912
BMI	26.2 (5.4)	24.8 (6.0)	0.239
Preoperative weight	76.6 (15.0)	66.1 (14.4)	0.001
Height	171.2 (10.2)	163.6 (5.7)	<0.001
ASA			
1	3 (4.5)	4 (11.4)	
2	34 (50.7)	21 (60.0)	
3	27 (40.3)	9 (25.7)	
4	3 (4.5)	1 (2.9)	
Chemotherapy			
Neoadjuvant	12 (18.2)	4 (11.4)	
Adjuvant	37 (56.9)	23 (65.7)	
Pathologic tumor stage			
II	21 (31.4)	13 (37.2)	
III	25 (37.3)	12 (34.3)	
IV	21 (31.3)	10 (28.6)	
Charlson Comorbidity Index	1.2 (1.8)	0.4 (0.7)	0.006
CCI	9.0 (14.7)	7.7 (15.7)	0.664
Preoperative sarcopenia markers			
IMAT area (SD)	18.2 (13.1)	16.7 (10.2)	0.541
IMATI (SD)	6.4 (5.5)	6.3 (3.9)	0.863
SMA (SD)	141.9 (29.0)	103.6 (17.4)	<0.001
SMI (SD)	48.4 (9.4)	38.9 (7.7)	<0.001
SMRA (SD)	37.7 (11.5)	37.6 (8.9)	0.981

**Table 2:** Cox’s regression analysis for overall survival—entire cohort. CH: chemotherapy; CCI: comprehensive complication index; SMA: skeletal muscle area; SMI: skeletal muscle index; SMRA: skeletal muscle radiation attenuation; IMAT: intermuscular adipose tissue; IMATI: IMAT index.

Variable	Coefficient (SE)	HR (95% CI)	*p*-Value
**Age**	**0.07 (0.03)**	**1.07 (1.00–1.14)**	**0.045**
Sex	−0.80 (0.79)	0.45 (0.10–2.11)	0.309
**ASA**	**0.88 (0.45)**	**2.41 (1.01–5.80)**	**0.048**
Neoadjuvant CH	0.84 (0.80)	2.31 (0.48–11.10)	0.297
Adjuvant CH	0.99 (0.71)	2.70 (0.67–10.78)	0.161
Charlson Comorbidity Index	0.22 (0.15)	1.24 (0.93–1.67)	0.146
CCI	0.02 (0.02)	1.02 (0.10–1.06)	0.312
**Δ IMAT (0–12)**	**−0.24 (0.09)**	**0.79 (0.67–0.93)**	**0.005**
**Δ IMATI (0–12)**	**−0.72 (0.26)**	**0.49 (0.30–0.80)**	**0.005**
Δ SMA (0–12)	−0.02 (0.03)	0.98 (0.92–1.05)	0.618
Δ SMI (0–12)	−0.05 (0.10)	0.95 (0.78–1.16)	0.626
Δ SMRA (0–12)	0.15 (0.10)	1.17 (0.96–1.42)	0.127

**Table 3:** Cox’s regression analysis of preoperative sarcopenia markers—male sex. BMI: body mass index.

Variable	Coefficient (SE)	HR (95% CI)	*p*-Value
Preoperative IMAT area	0.02 (0.02)	1.02 (0.98–1.07)	0.307
Preoperative IMATI	0.03 (0.05)	1.03 (0.94–1.14)	0.498
Preoperative SMA	−0.02 (0.01)	0.98 (0.96–1.00)	0.097
Preoperative SMI	−0.07 (0.05)	0.93 (0.85–1.02)	0.128
Preoperative SMRA	−0.06 (0.04)	0.94 (0.87–1.01)	0.078
BMI	0.04 (0.05)	1.04 (0.93–1.15)	0.496

**Table 4:** Cox’s regression analysis of preoperative sarcopenia markers—female sex.

Variable	Coefficient (SE)	HR (95% CI)	*p*-Value
Preoperative IMAT area	0.04 (0.06)	1.04 (0.92–1.18)	0.504
Preoperative IMATI	0.11 (0.17)	1.11 (0.80–1.55)	0.522
Preoperative SMA	0.03 (0.04)	1.03 (0.95–1.12)	0.454
Preoperative SMI	0.05 (0.08)	1.06 (0.90–1.24)	0.511
Preoperative SMRA	−0.27 (0.16)	0.76 (0.56–1.05)	0.094
BMI	0.18 (0.11)	1.20 (0.96–1.51)	0.109

## Data Availability

The data gathered in this study is available upon reasonable request to the authors for ethical reasons.
